# The most important things I have learnt in my career as a psychiatrist

**DOI:** 10.1192/pb.bp.115.052092

**Published:** 2017-02

**Authors:** Julian Leff

**Affiliations:** 1 Institute of Psychiatry, Psychology and Neuroscience, King's College London

## Abstract

This paper is something of a patchwork, incorporating many issues that have intrigued me during 34 years of research. I have included the importance of maintaining a solid base in clinical work, alongside research activities, and being alert to the possibility of a somatic condition contributing to psychiatric symptoms. I stress the value of careful observation of patients, their response to treatments and reasons for dropping out. In addition, I have included 14 more lessons, learned from my experience of research, which I hope will be of use to those readers who aspire to become researchers.

I was attracted to psychiatry by my experience as a medical student at University College Hospital. Students on the psychiatry firm were expected to take on a psychotherapy patient, supervised by one of the senior therapists. This was the first time in my medical training that I felt valued as a clinician and treated as a responsible adult. (There is an important lesson here for improving the very low recruitment of medical students to psychiatry.)

Having graduated as a doctor I was keen to begin my study of psychiatry and sought advice from Sir Martin Roth, president of the Royal College of Psychiatrists at that time. He was adamant that I had to obtain the Membership of the Royal College of Physicians before applying for training in psychiatry. This was daunting as the Physicians' exam had a very low pass rate. I spent 3 arduous years as house officer in hospitals in London's East End before passing the exam. However, in a strange way it felt like coming home. This was because my father and his family lived in a house in the East End, at least until it was completely destroyed by a German bomb. By working with East Enders I learned to appreciate the fortitude and strength of people in the lowest stratum of society, and I never forgot that.

Throughout my career I always continued with clinical work alongside my role as director of two research teams, holding weekly out-patient clinics and having consultant responsibility for half an acute admission ward at the Maudsley Hospital and later at Friern Hospital. In addition, I supervised community mental health teams for their work with families. In this paper I will present the important things I have learned from both my clinical and my research activities.

Becoming a Fellow of the Royal College of Physicians (FRCP), I applied to the Maudsley training scheme and was accepted. When I met the other trainees in my intake, I found to my chagrin that only half of them had passed the Physicians' exam. There was one occasion in my psychiatric practice when my medical expertise proved its value. An out-patient in my clinic was complaining of anxiety and breathlessness, specifically when leaving her home and being in crowds. Naturally, I diagnosed a phobic condition and prescribed an anxiolytic. As she was about to leave the clinic, she said, ‘By the way, doctor, I have a rash on my legs’. I asked her to show it to me and immediately recognised the raised purplish bumps of erythema nodosum. I referred her to the medical specialists who took over the treatment of her medical condition and cured her of her breathlessness.

## Lesson 1: always bear in mind the possibility of an underlying medical condition

There is an expanding literature on comorbidity between psychiatric and medical conditions that partly contributes to the high mortality rate in psychiatric patients.

## Lesson 2: make careful observations on patients and their response to treatments

Observant clinicians remain curious about puzzling aspects of everyday psychiatry, and this attitude can stimulate research projects which have the potential to lead to breakthroughs in psychiatric treatments. For example, my concern about the failure of antipsychotic medication to suppress auditory hallucinations in one in four patients led me to develop avatar therapy,^1^ a novel and effective treatment for this debilitating condition.

## Lesson 3: always listen carefully to the patient's narrative

Do not dismiss the patient's belief about the cause and nature of their illness. It has been shown that the greater the disparity between the patient's and the physician's understanding of the patient's illness, the less likely the patient is to adhere to the prescribed treatment. The same caveat applies to the family carers' beliefs about the patient's illness, even if they depart widely from your own understanding. UK psychologists were the first to adapt Beck's cognitive therapy for depression to treat psychosis (CBTp). My experience of working with family carers of people with schizophrenia taught me that many low expressed emotion family carers had developed effective coping strategies for dealing with the patient's difficult behaviour, for example, distraction, reality testing and time out, which closely resembled CBTp. If professionals had listened to these family carers, and given credence to their narratives, they would have introduced CBTp much earlier, saving considerable suffering. While CBTp alleviates the anxiety of patients with persecutory auditory hallucinations, it has little impact on their frequency or volume, as opposed to avatar therapy.^1^

## Lesson 4: the value of qualitative studies

Qualitative studies provide information of a different nature to quantitative studies, such as patients' experience of novel therapies, but they are of equal importance. Quantitative and qualitative studies are not in competition, but complement each other and are very informative when employed in tandem. An example is the current large-scale replication study of the value of avatar therapy.^2^ Standard quantitative measures of the participants' assessment of the power and malignancy of their persecutory voices are augmented by the participants' accounts of their experience of standing up to the avatar they have created as a proxy for their persecutor.

## Lesson 5: it is all too easy to become fascinated with esoteric research and to allow its demands to divert you away from basic clinical work

Working with patients is the crucible that generates the questions that inspire research projects. I cannot emphasise enough the importance of maintaining contact with everyday psychiatric patients, and not being lured exclusively by the glamour of exotic conditions. When I was granted a chair, I was the only professor in the Institute of Psychiatry (now Institute of Psychiatry, Psychology and Neuroscience (IoPPN), King's College London) who held clinics for local patients. Happily, that situation has now changed.

My research career began in earnest in 1968 when I was offered a position in John Wing's Medical Research Council Social Psychiatry Unit at the Institute of Psychiatry. I spent 34 years as a researcher, focusing mainly, but not exclusively, on psychosis. Many of the important lessons I learned were as a result of my research experiences, and it is these I wish to share with you, in the hope that they will inspire you to undertake your own research projects. There are so many unanswered questions in psychiatry that we need many more researchers to tackle these. Sir Aubrey Lewis, who founded the Institute of Psychiatry, was dedicated to promoting research and insisted that trainees conduct their own research project in order to pass their final exam. Sadly this requirement lapsed after Sir Aubrey retired.

## Lesson 6: formulating a question

Junior colleagues would often approach me, eyes shining with excitement, saying that they wanted to do research ‘with a capital R’. I would then ask them what they would like to do their research on and would be met by a baffled silence, clearly expecting me to spell out a project for them. If a topic engages your curiosity, you need to do some hard thinking and formulate a question that you want to answer. A well-constructed question can be developed into a series of hypotheses, which will determine the trajectory of your research project. A well-designed research study will always generate new unanswered questions that will then form the basis for the next study. The results of a research project you did not expect are as important as those you anticipated, and should lead you on to a new study. Consider the following sequence.

When Christine Vaughn joined the Social Psychiatry Unit, she decided that she wanted to embark on a PhD to replicate the findings of Brown, Birley and Wing that high expressed emotion in family carers significantly predicted relapse of schizophrenia.^3^ This pioneering study was surprisingly neglected by the psychiatric community, probably because of the prevailing adherence to biological explanations for schizophrenia. Christine and I decided that a replication would attract the attention these findings deserved.

We discussed collaborating on this project and I suggested that it would add value to the research if we included a group of patients with depressive disorder to ascertain whether expressed emotion was a specific predictor for schizophrenia or whether it would predict relapse of depression as well. In the event it turned out to be a more potent predictor for relapse of depression than for relapse of schizophrenia, as shown by Jill Hooley in her PhD in the USA.^4^ In addition, our study replicated the protective effects of medication and low contact with the carer against the stress of high expressed emotion that the earlier study had revealed. This stimulated me to consider the possibility of intervening in high expressed emotion families to lower expressed emotion and contact between the patient and the family carers. There were two compelling reasons for this: primarily, to determine the direction of causality (do high expressed emotion attitudes cause relapse of schizophrenia or does the patient's disturbed behaviour provoke high expressed emotion attitudes?); and also to determine whether our family interventions could improve the outcome of schizophrenia.

For this project I assembled a team of therapists with different areas of expertise: a cognitive-behavioural therapist with experience of working with groups, a psychodynamic psychiatrist, a psychiatrist from Germany who was a trained psychoanalyst and myself as an eclectic therapist with no strong adherence to any one theory of family functioning. The therapy used by this heterogeneous team was remarkably effective, succeeding in reducing the patients' relapse rate over 9 months from 50% to less than 10%.^5^ The success of this trial led to two more randomised controlled trials (RCTs), including a cost-benefit study. The accumulation of this evidence plus ten replications in different countries led the National Institute for Health and Clinical Excellence (NICE) to recommend that family carers of a patient with schizophrenia must have professional input. This outcome justified the 15 years it took to complete the sequence of RCTs.

## Lesson 7: the danger of linear thinking

Linear thinking results from the idea that causal action flows in one direction only, from cause to effect, from producer to produced, analogous to a series of billiard balls in motion. The issue of linear thinking in the social sciences, including psychiatry, merits some discussion.

Linear thinking forms the basis of almost all biological research in psychiatry. It has a long history, having been first formulated by the Greek philosophers Anaximander and Plato. In the modern era, Von Bertalanffy was the first to challenge linear thinking.^6^ He stated that we must think in terms of systems of elements in mutual interaction. At that time, the development of cybernetics promoted systems thinking. The process of feedback whereby missiles could monitor and correct their trajectory was recognised as analogous to the biological system's capacity to maintain and organise itself in nature. This formulation was seen as very relevant to the process of family therapy. The family theorist Gregory Bateson wrote: ‘I think that cybernetics is the biggest bite out of the Tree of Knowledge that mankind has taken in the last 2000 years’.^7^ It is ironic that a technology developed to destroy human beings should contribute to our understanding of family relationships.

Homeostasis is a concept fundamental to systems theory. It is achieved by negative feedback loops, which stabilise the system by reducing deviation between goal and performance (cf. missiles). By contrast, positive feedback loops reinforce or amplify deviations, producing novelty and instability and an increase in the complexity of the system, leading to new properties. Applying this understanding to social relationships, which are of central importance to psychiatry, feedback represents the direct perceptual report of the effect of one's behaviour on others, for example, the perception of a smile in response to one's own smile.

Robert Dubin considers that the difficulty of avoiding linear thinking stems from our propensity to look for isolatable one-way causes.^8^ Feedback processes can easily be overlooked, not only because the linear perspective is the dominant mode, but also because they tend to be unnoticed owing to their very pervasiveness.

Earlier I stated that one compelling reason for working with high expressed emotion families was, I quote myself, ‘to determine the direction of causality’. You will now recognise this as a prime example of linear thinking. In actuality I was aware that there were multiplex interactions between patients and their family carers, but this was too complex to investigate at that time.

Now I will give you some practical advice on initiating research and carrying it through to publication.

## Lesson 8: searching the literature

Electronic databases have made this much simpler and more efficient. Decide on the criteria for your search, and be overinclusive rather than underinclusive (obviously, irrelevant papers can be deleted without needing to read them). Summarise what has been established. This requires a critical attitude to research by others, however eminent they may be. Weigh up the evidence and come to a conclusion. This may be that the question you formulated has been adequately answered, in which case, back to the drawing board!

## Lesson 9: focus on a single topic

Avoid being too ambitious. If your initial project produces useful results, you can always extend it. Seek advice from experienced colleagues. They can warn you about pitfalls in your chosen area of research.

## Lesson 10: applying for funding

When applying for funding, choose the funding body carefully, paying close attention to their mission statement. It is often worthwhile beginning with a pilot study which can be mounted with minimal or no costs. For instance, determining whether your catchment area will provide sufficient patients for your study. This will show potential funders that you are a serious contender.

## Lesson 11: anticipate the concerns of the ethics and research and development committees

Gaining approval from these committees is now an obligatory hurdle to surmount. There are a number of actions you can take to improve your chances of being approved. Anticipate objections from committee members, and be prepared to be able to counter them. In my recent trial of avatar therapy, I anticipated that there would be anxiety in the committee about patients' response to being faced with their persecutor in the shape of the avatar. Therefore, with the aid of my IT specialist, we constructed a bright red ‘stress button’ which the patient could press in case of high anxiety or for any other reason. This switched off the avatar image on the monitor, which was replaced by an image of a tropical beach with *The Four Seasons* by Vivaldi playing in the background. In the event, only 2 patients out of 18 pressed the stress button, and both were able to continue with the session after reassurance.

## Lesson 12: appreciate the value of user-researchers

Involve service users in your study. They should certainly be asked to read the instructions for potential participants, and to suggest changes to the wording. Users can be recruited to play a more important role in your study. The IoPPN has established a list of user-researchers. These are users who have largely recovered from a psychiatric illness and are willing and able to be trained in research procedures. For example, in my avatar therapy trial, I employed a user-researcher who had heard voices himself 8 years previously and was now completely well. I trained him in the assessment tools and he achieved high interrater reliability with me, enabling him to undertake the role of an independent assessor, for which of course he was paid. The employment of users will be greatly appreciated by the ethical committee. If you do appoint a user-researcher, they must be included as an author.

## Lesson 13: team building

If you are ambitious and wish to undertake a major study you will need a team, preferably multidisciplinary in nature. Diversity of professional expertise is an asset, as we experienced in our development of working with families. Consult a statistician early on in designing the study. Statisticians are understandably grumpy if they are asked at the last minute to conduct the data analysis without having given any prior input. Develop a cohesive group and deal with rivalry. The media often depict research as a gentlemanly pursuit of the truth. That is a fallacy. There are glittering prizes to be won through research, and the world of research is as competitive and cut-throat as multinational capitalism.

Be fair to junior members of the team. Encourage and support them and give them experience in presenting and appropriate representation in publications. In mid-career I left the Maudsley to take charge of a dysfunctional research group working in a traditional psychiatric hospital. It had been managed by two absentee directors and was in a state of anarchy, with one member of staff suing the directors. The aggrieved staff member left and I had to dismiss another member of staff. I knew my intervention would be resented so introduced a Friday lunch-time picnic in the extensive grounds of the old psychiatric hospital, followed by a game of croquet on the lawn next to the former medical superintendent's villa. I reasoned that being able to knock my balls around would diffuse aggression, and so it did.

There were two reasons for my leaving the Maudsley to work in Friern, a typical 19th-century asylum. One was to emerge from the shadow cast by John Wing, the director of the Medical Research Council Social Psychiatry Unit. Although John left me to pursue my own research interests, he was nearing retirement and I knew that to stand a chance of taking over the directorship of the Unit, I would have to prove that I was capable of mounting important research independently from him. The other reason was the split in the profession of psychiatry between the academics and psychiatrists working in provincial hospitals; the latter felt overburdened by their workload and disregarded by the academics, whom they saw as existing in a ‘cloud 9’ environment, protected from the realities of jobbing psychiatry. Given that in that era the great majority of psychiatrists were working in antiquated buildings, with insufficient support from psychologists, occupational therapists and other ancillary staff, I felt that I needed to experience the reality of life in an asylum.

It felt to me like another world. The Italianate Gothic frontage was forbidding, as was the original plaque designating the building as the West Sussex Pauper Lunatic Asylum. The entrance corridor was a third of a mile long. At that time it was the longest hospital corridor in Europe. It had windows throughout its length, but they were so low I could only see through them by stooping uncomfortably. At the end of the corridor was a faint glimmer of light from the world outside. Despite the gloom that descended on me, in time I began to appreciate the good qualities of Friern. It was set in extensive grounds, which included a football field and a 9-hole golf course, on which I never saw anyone playing. There was a chapel and a synagogue, and a factory outlet where low-cost clothes were available. Although the main gate was always open, very few patients ventured out into the street. Patients wandered around the grounds unhindered and sexual liaisons were undoubtedly formed, as one of the long-stay patients in my care regularly developed gonorrhoea. I began to appreciate how easy it would be to become accustomed to the environment of the asylum and to forget the existence of the outside world.

## Lesson 14: the staff in an institution are often more institutionalised than the patients

Not long after I moved to Friern Hospital the Regional Health Authority decided to close Friern and Claybury hospitals in accord with the government policy of that time. I realised that this was a unique opportunity to evaluate this policy. I succeeded in obtaining funding from the Regional Health Authority, later supplemented by funding from the Department of Health. This enabled me to form a group of researchers under the title of TAPS: The Team for the Assessment of Psychiatric Services. Friern Hospital had been opened in 1851 with 1000 beds. The number of patients grew exponentially, reaching 2500 in the 1940s. The discharges of many patients between 1940 and the beginning of the TAPS programme had reduced the number of long-stay patients to 800.

The first step in the TAPS project was to conduct a comprehensive assessment of the symptoms and the problem behaviours of all the remaining patients in the two hospitals who did not have dementia. Complete data on all 700 patients were collected by the team, a mammoth undertaking. A 5-year follow-up was conducted on this group of patients, of whom only a tiny number were lost to the study, thanks to the efforts of the excellent administrative assistant who made regular checks on the patients' locations in the community. Meanwhile, an extraneous researcher, not a TAPS member, carried out a survey of all the nursing staff looking after the remaining patients, asking them to estimate the number of patients who could be resettled in the community. The total percentage estimated by the nursing staff was one-third. If this was accurate, the possibility of closing the two hospitals within the 10-year limit set by the managers was negligible. However, the TAPS team had already begun asking individual patients for their preferences when the hospitals closed: one-third wanted to leave the hospital and live in the community, one-third opted to stay in the hospital and one-third were uncertain. In the event, all the patients considered suitable to live in the sheltered homes in the community by the resettlement teams adapted well to life in the outside world, and when asked where they would prefer to live a year later, 84% wanted to stay where they were.^9^

Friern hospital did close on time 10 years after the decanting began.The Claybury closure was delayed because the consultants there went on strike against the closure decision, but the strike collapsed after 6 months and the closure went ahead. So psychiatrists can be as institutionalised as nursing staff.

## Lesson 15: presenting at conferences

Always try out your presentations with a sympathetic audience and take note of their criticisms and comments. The golden rule for slides is ‘never put more on a slide than you can get on a T-shirt’. I am often amazed at seeing experienced researchers cramming a slide with illegible lists of data and *P*-values and then saying to the audience, ‘You probably won't be able to read this but what it shows is … ’ – if it can't be read, don't show it!

## Lesson 16: writing up the results

Avoid the pall of conventional scientific writing. Break through the conventional anonymity of the passive voice. Humanise your writing to make it attractive to the reader. Keep the language simple and avoid too many technical terms. Always spell out abbreviations the first time they appear in a paper, including the abstract. Pay special attention to the clarity and layout of tables and figures – ensure that they are essential for the understanding of your results. Editors dislike large numbers of tables and figures since they occupy space that could be used to publish another paper. Avoid duplicating results in the text as well as in tabular form.

## Lesson 17: submission to a journal

Choose the journal carefully, surveying past issues for the types of papers published. Always read instructions to authors with great care and observe them, particularly the word limit – if you exceed this, your paper will bounce back rapidly. Find out the proportion of submissions accepted, if possible. Always treat the reviewers' comments seriously and couch your responses respectfully, even if you think the reviewers are idiots. Don't give up at the first rejection, but look for alternative journals. There are so many journals being published now that there is considerable overlap in their remit. If you are inexperienced, do not be too ambitious in choosing a journal with a high impact factor. I sent my recent paper on avatar therapy serially to *Nature, New Scientist* and *Archives of General Psychiatry*, all of which rejected it without sending it for review. So much for hubris! It was eventually published in the *British Journal of Psychiatry*. I was mollified when following a press conference the paper went global.

## Maintaining safety

I am interpolating this issue, not to raise your anxieties, but to convey the important advice I received from a senior colleague early in my clinical career. What he said was ‘never let the patient get between you and the exit door of your clinic’. A colleague and friend of mine at the Maudsley Hospital was unaware of this advice and preceded the patient into his clinic; the patient then stabbed him in the back with a pair of scissors. Fortunately, the wound was superficial.

## Finale

Do not be put off by the hard work and inevitable disappointments. They are more than compensated for by the intellectual excitement generated by research and the knowledge that you are improving the lives of your patients.
